# Electron-Impact Cross Sections for Dipole- and Spin-Allowed Excitations of Hydrogen, Helium, and Lithium

**DOI:** 10.6028/jres.107.026

**Published:** 2002-08-01

**Authors:** Philip M. Stone, Yong-Ki Kim, J. P. Desclaux

**Affiliations:** National Institute of Standards and Technology, Gaithersburg, MD 20899-8421; 15 Chemin du Billery, F-38360 Sassenage, France

**Keywords:** electron-impact, excitation cross section, hydrogen, helium, lithium

## Abstract

Electron-impact excitation cross sections are presented for the dipole- and spin allowed transitions from the ground states to the *np* ^2^P states for hydrogen and lithium, and to the 1*snp* ^1^P states for helium, *n* = 2 through 10. Two scaling formulas developed earlier by Kim [Phys. Rev. A **64**, 032713 (2001)] for plane-wave Born cross sections are used. The scaled Born cross sections are in excellent agreement with available theoretical and experimental data.

## 1. Introduction

We have scaled Plane-Wave Born (PWB) cross sections to calculate dipole- and spin-allowed excitation cross sections from the ground state of neutral hydrogen, helium, and lithium. The scaling method was developed by one of us [1], and uses two simple scaling formulas to convert PWB excitation cross sections into reliable cross sections comparable to the most accurate theoretical or experimental data available for dipole-allowed transitions. The PWB cross sections are calculated from uncorrelated wave functions, and the scaling requires only the binding energy *B* of the electron being excited, the excitation energy *E*, and an accurate dipole oscillator strength *f* for the transition. The oscillator strength is needed only if electron correlation strongly affects the *f* value, i.e., when the wave functions used to calculate the PWB cross section are not accurate. Simplicity of the method to scale PWB cross sections allows us to generate a large number of cross sections reliably and quickly.

In this paper, we present calculated excitation cross sections for hydrogen from the 1*s* ^2^S ground state to the *np* ^2^P excited states. For helium, the cross sections are given for excitations from the 1*s*^2 1^S ground state to the 1*snp* ^1^P excited states. For lithium, the results are given for excitations from the 1*s*^2^2*s* ^2^S ground state to the 1*s*^2^*np* ^2^P states. In all cases, the values of *n* are from *n* = 2 through 10.

## 2. Outline of Theory

A PWB cross section for electron-impact excitation, *σ*_PWB_, has the form

$$\sigma_{\text{PWB}}=\frac{4\pi a_{0}^{2}R}{T}\;F_{\text{PWB}}(T),$$ (1)

where *T* is the incident electron energy, *a*_0_ is the Bohr radius (0.529 Å), and *R* is the Rydberg energy (13.61 eV). The *F*_PWB_(*T*) is the collision strength (different from the standard definition by a multiplicative constant).

The first scaling method, BE scaling, replaces *T* in the denominator of Eq. (1) by *T* + *B* + *E*, i.e.,

$$\sigma_{\text{BE}}=\sigma_{\text{PWB}}\;[T/(T+B+E)].$$ (2)

This scaling is similar to a scaling for ionization cross sections used earlier by Burgess [2], who shifted the incident energy *T* by *B*+*U*, where *U* is the kinetic energy of the target electron. However, in the BE scaling adopted by Kim [1] for excitation cross sections, *T* is shifted by *B*+*E*. The BE scaling not only changes the magnitude but also the shape of the original PWB cross sections. The BE scaling corrects the deficiency in the collision theory; i.e., the use of the PWB approximation.

The second scaling formula, the *f* scaling, multiplies the entire cross section by the ratio of an accurate *f* value to the less accurate *f* value calculated by the actual wave functions used to generate the unscaled PWB cross sections:

$$\sigma_{f}=(f_{\text{accu}}/f_{\text{sc}})\sigma_{\text{PWsc}}.$$ (3)

where *f_sc_* is the single configuration (or uncorrelated) *f* value and *f*_accu_ is the more accurate value obtained from correlated (or multiconfiguration) wave functions or from a reliable experiment. Accurate *f* values are frequently available [3]. The *f* scaling compensates for the inadequacy of the wave functions when electron correlation effect is significant. The BE and *f* scalings may be applied consecutively, i.e.,

$$\sigma_{\text{BE}f}=(f_{\text{accu}}/f_{\text{sc}})\sigma_{\text{BEsc}},$$ (4)

where *σ*_BEsc_ is the BE-scaled PWB cross section calculated from single-configuration wave functions.

Kim has shown many examples [1] in which the BE scaling alone or in combination with the *f* scaling transformed PWB cross sections for dipole-allowed and spin-allowed excitations into reliable cross sections comparable to the convergent close coupling (CCC) method [4] or accurate experiments.

In reality, electron-impact excitation cross sections of atoms have resonances in the vicinity of the excitation thresholds caused by the formation of transient compound states between the incident electron and the target atom. First-order perturbation theories such as the PWB approximation cannot account for such compound states, and hence the present scaled cross sections do not exhibit any resonances.

The numerical data in Tables 1, 2, and 3 can easily be extended to higher incident energies by using the well known Bethe formula [5] for the plane-wave Born approximation for fast (but nonrelativistic) incident electrons. In our notation, the asymptotic expression becomes:

$$\sigma_{\text{asympt}}(T)=\frac{4\pi a_{0}^{2}R}{T+B+E}[a\;\text{ln}(T/R)+b+cR/T](f_{\text{accu}}/f_{\text{sc}}),$$ (5)

where *a, b*, and *c* are dimensionless constants. Equation (5) should be used for *T* > 3 keV. The values of *a*, *b*, and *c* are included in Tables 1, 2, and 3. Note that a relativistic form (5) of Eq. (5) should be used for *T* > 10 keV.

## 3. Theoretical Results

**Fig. 1:**
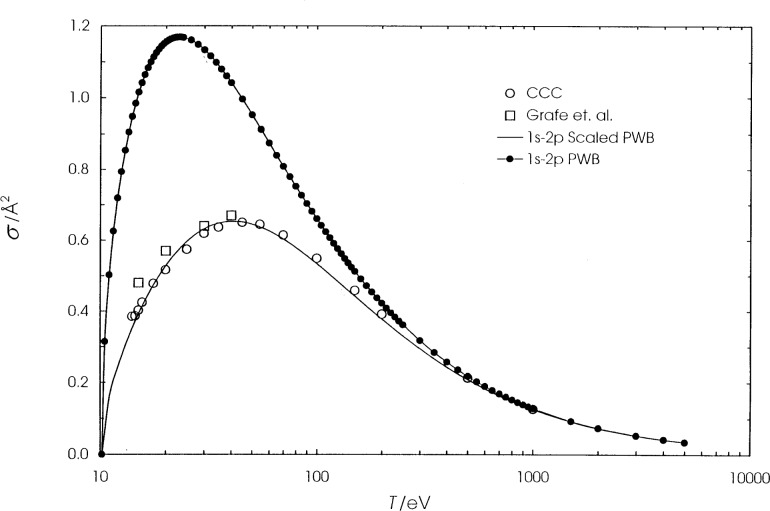
Hydrogen 1*s*–2*p* electron–impact excitation cross sections. The solid curve is our scaled plane–wave Born (PWB) result, the filled circles are unscaled PWB cross sections, the open circles are accurate theoretical results from the convergent close coupling (CCC) method shown on the web site of Bray and Ralchenko [6], and the squares are experimental results of Grafe et al. [11].

**Fig. 2:**
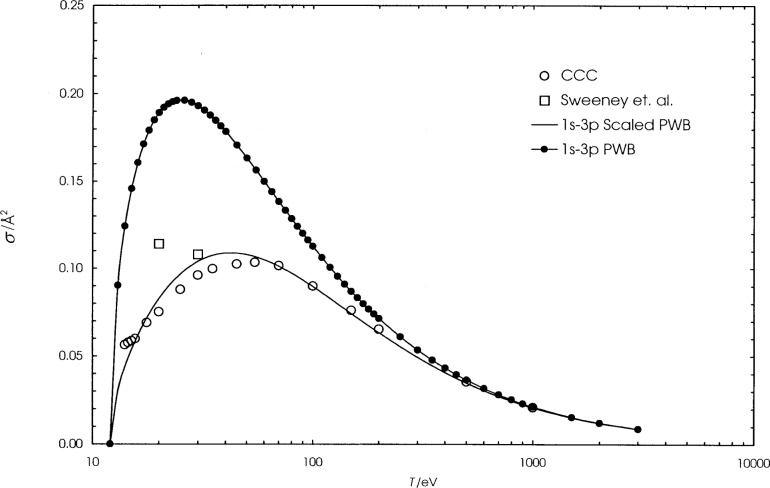
Hydrogen 1*s*–3*p* electron–impact excitation cross sections. The squares are experimental results of Sweeney et al. [7]. The other results are as in Fig. 1.

**Fig. 3:**
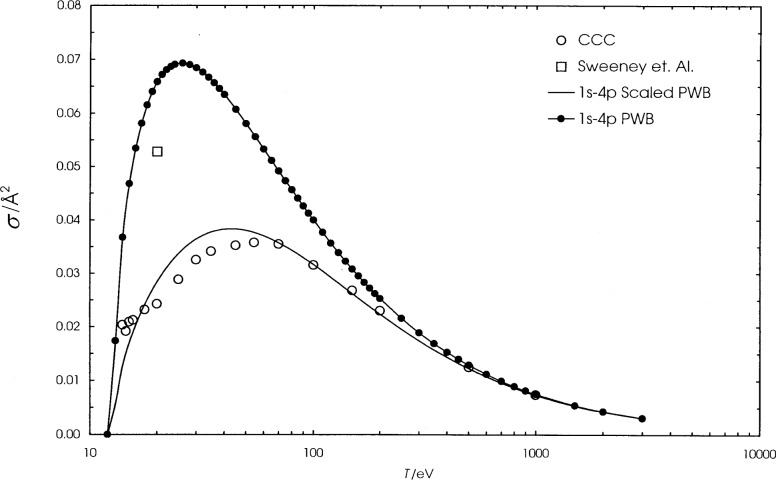
Hydrogen 1*s*–4*p* electron–impact excitation cross sections. The symbols are as in Fig. 2.

**Fig. 4:**
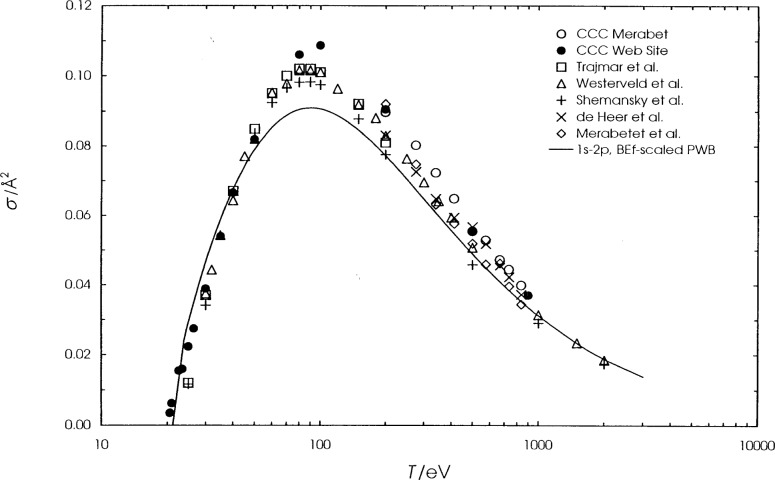
Helium 1*s*^2 1^S–1*s*2*p* ^1^P electron–impact excitation cross sections. The solid curve is the BE*f*–scaled plane–wave Born cross section, the open circles are recent CCC results of Merabet et al. [12], and the filled circles are previous CCC results from the web site of Bray and Ralchenko [6]. Experimental results are the recommended values of Trajmar et al. [16] (open squares), Westerveld et al. [14] (triangles), Shemansky et al. [15] (pluses), recommended values of de Heer et al. [13] (crosses), and results of Merabet et al. [12] (diamonds).

**Fig. 5:**
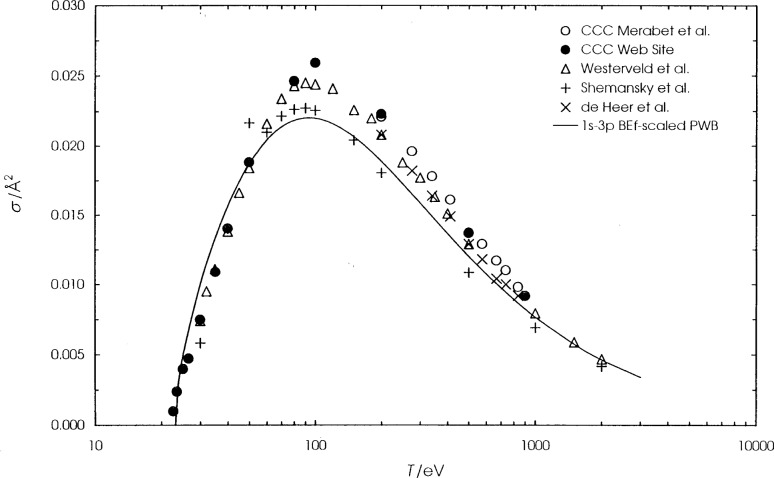
Helium 1*s*^2 1^S–1*s*3*p* ^1^P electron–impact excitation cross sections. Symbols are the same as in Fig.4.

**Fig. 6:**
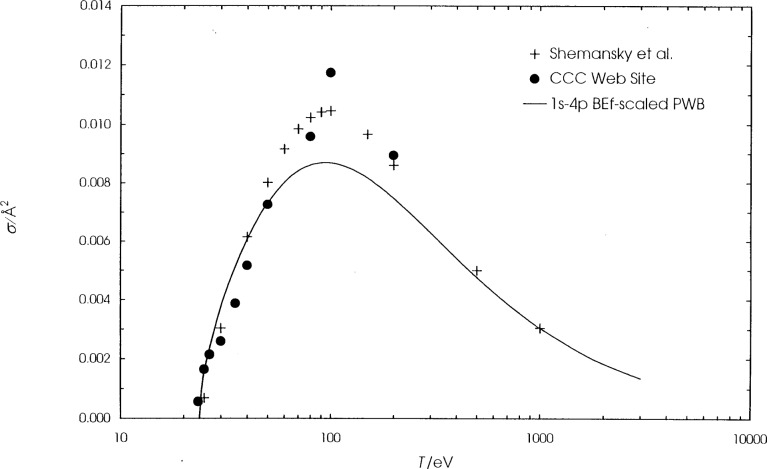
Helium 1*s*^2 1^S–1*s*4*p* ^1^P electron–impact excitation cross sections. Symbols are the same as in Fig.4.

**Fig. 7:**
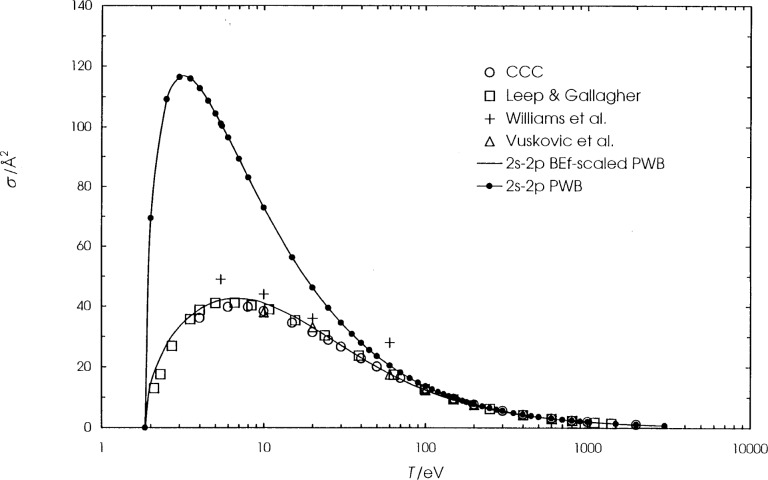
Lithium 1*s*^2^2*s*–1*s*^2^ 2*p* electron–impact excitation cross sections. The solid curve is the present scaled plane–wave Born (PWB) cross section, the filled circles are unscaled PWB cross section, and the CCC results (open circles) are from Schweinzer et al. [22]. Experimental results are from Leep and Gallagher [18] (open squares), Williams et al. [19] (pluses), and Vuskovic et al. [20] (triangles).

We present the calculated cross sections for hydrogen, helium, and lithium in Tables 1–3. Our PWB cross sections were generated from single configuration Dirac-Fock wave functions. The calculated cross sections are compared to other theories and experiments in Figs. 1–7.

The CCC results for these elements are from the web site of Bray [6]. The experimental results by Sweeney et al. [7] for hydrogen include all dipole-allowed and dipole-forbidden states of hydrogen for each *n*, and hence are higher than the cross sections for just the dipole-allowed excitations.

**Fig. 8:**
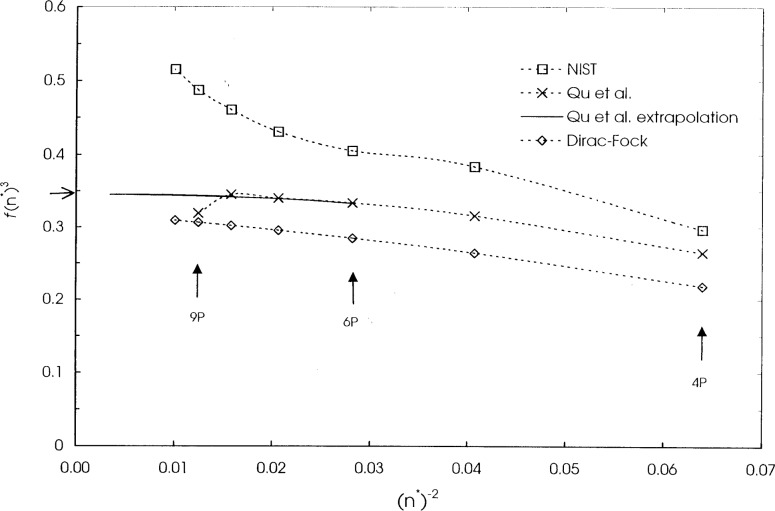
Values of *f*(n^*^)^3^ for lithium showing the extrapolated values for Qu et al. [10] from their data for *n* = 6 and 7. The *f*–values given by Qu et al. are anomalous at *n* = 8 and 9. The extrapolated value at the ionization limit (n^*^ → ∞) is 0.345 (indicated by the arrow on the left hand axis). Also shown are the results from the single configuration Dirac–Fock calculation of the present work (diamonds) and values from the current NIST web site [3] (squares). The NIST web site values are earlier results than the more accurate data of Qu et al.

The ionization energies *B* and the excitation energies *E* are all experimental values. Only BE scaling is needed for hydrogen as its exact wave functions are known. The accurate *f* values for helium have been obtained from the detailed variational calculations of Drake [8]. The *f* value for the 2*s*–2*p* transition in lithium is from the calculations of Yan et al. [9], while the values for the 2*s*–*np* transitions, *n* = 3 through 7, are from the non-relativistic multiconfiguration calculations including core polarization by Qu et al. [10]. For the 2*s*–8*p*, 9*p*, and 10*p* excitations of lithium, we extrapolated *f*(n*)^3^ of Qu et al. [10] from *n* = 5 through *n* = 7, where n* is the experimental effective principal quantum number of quantum defect theory. We had found that the *f* values by Qu et al. for the 8*p* and 9*p* transitions were inconsistent with their values for *n* < 8. The extrapolation of *f*(n*)^3^ is shown in Fig. 8 through n* ≈ 17 and is given by the expression:

$$f{\left(n*\right)}^{3}=0.343+0.0283/(n*)+0.533/{\left(n*\right)}^{2}-6.289/{\left(n*\right)}^{3}.$$ (6)

Beyond n* ≈ 17, the formula begins to break down but the actual curve should remain flat. At the ionization limit (n* → ∞), the value of *f*(n*)^3^ is extrapolated to be 0.345.

It is apparent that for all cases where experimental data and CCC results are available, the scaled PWB cross sections give values that are in good agreement.

## Figures and Tables

**Table 1:** Hydrogen. Excitation energies *E* in eV, dipole *f* values, and BE–scaled excitation cross sections σ_BE_ in Å^2^ as functions of incident electron energy *T* in eV. The experimental ionization energy *B* = 13.5984 eV has been used in the scaling. The constants *a*, *b*, and *c* of Eq. (5) are included

Excitation	1*s*–2*p*	1*s*–3*p*	1*s*–4*p*	1*s*–5*p*	1*s*–6*p*	1*s*–7*p*	1*s*–8*p*	1*s*–9*p*	1*s*–10*p*
*E*	10.204	12.094	12.755	13.061	13.228	13.328	13.393	13.438	13.470
*f*	0.4164	0.0791	0.0290	0.0139	0.00780	0.004816	0.003185	0.002217	0.001606
Const.*a*	0.555512	0.089083	0.030956	0.014534	0.008031	0.004919	0.003237	0.002246	0.001623
Const. *b*	0.271785	0.060202	0.022984	0.011243	0.006348	0.003939	0.002550	0.001824	0.001323
Const. *c*	0.000112	−0.019775	−0.009279	−0.004880	−0.002853	−0.001806	−0.001213	−0.000854	−0.000623
*T*	*σ*_BE_	*σ*_BE_	*σ*_BE_	*σ*_BE_	*σ*_BE_	*σ*_BE_	*σ*_BE_	*σ*_BE_	*σ*_BE_
11	0.15876								
12	0.24099								
13	0.30186	0.03033	0.00573						
14	0.35119	0.04382	0.01274	0.00528	0.00267	0.00154	0.000965	0.000646	0.000455
15	0.39256	0.05372	0.01697	0.00752	0.00401	0.00240	0.00155	0.00106	0.000763
16	0.42786	0.06166	0.02018	0.00916	0.00496	0.00300	0.00195	0.00135	0.000969
17	0.45828	0.06827	0.02278	0.01046	0.00570	0.00346	0.00227	0.00157	0.00113
18	0.48468	0.07387	0.02496	0.01154	0.00632	0.00385	0.00252	0.00175	0.00126
19	0.50768	0.07867	0.02681	0.01245	0.00684	0.00417	0.00274	0.00190	0.00137
20	0.52779	0.08282	0.02840	0.01323	0.00728	0.00445	0.00292	0.00202	0.00146
21	0.54540	0.08642	0.02977	0.01391	0.00766	0.00468	0.00308	0.00213	0.00154
22	0.56084	0.08957	0.03096	0.01449	0.00799	0.00489	0.00321	0.00223	0.00161
23	0.57439	0.09231	0.03200	0.01500	0.00828	0.00507	0.00333	0.00231	0.00167
24	0.58627	0.09472	0.03291	0.01544	0.00853	0.00522	0.00344	0.00238	0.00172
26	0.60580	0.09867	0.03440	0.01617	0.00894	0.00548	0.00361	0.00250	0.00181
28	0.62069	0.10169	0.03554	0.01673	0.00925	0.00567	0.00373	0.00259	0.00187
30	0.63189	0.10398	0.03641	0.01715	0.00949	0.00582	0.00383	0.00266	0.00192
32	0.64013	0.10569	0.03706	0.01747	0.00967	0.00593	0.00391	0.00271	0.00196
34	0.64597	0.10695	0.03754	0.01771	0.00981	0.00602	0.00396	0.00275	0.00199
36	0.64986	0.10782	0.03788	0.01787	0.00990	0.00608	0.00400	0.00278	0.00201
38	0.65216	0.10840	0.03811	0.01799	0.00997	0.00612	0.00403	0.00280	0.00202
40	0.65315	0.10872	0.03825	0.01806	0.01001	0.00614	0.00405	0.00281	0.00203
45	0.65130	0.10870	0.03828	0.01808	0.01002	0.00615	0.00405	0.00281	0.00204
50	0.64520	0.10787	0.03801	0.01796	0.00996	0.00611	0.00403	0.00280	0.00202
55	0.63647	0.10654	0.03756	0.01775	0.00984	0.00604	0.00398	0.00277	0.00200
60	0.62615	0.10490	0.03700	0.01749	0.00970	0.00595	0.00392	0.00273	0.00197
65	0.61489	0.10308	0.03636	0.01719	0.00953	0.00585	0.00386	0.00268	0.00194
70	0.60315	0.10116	0.03569	0.01688	0.00936	0.00575	0.00379	0.00263	0.00190
75	0.59121	0.09919	0.03500	0.01655	0.00918	0.00564	0.00371	0.00258	0.00187
80	0.57929	0.09721	0.03431	0.01622	0.00900	0.00552	0.00364	0.00253	0.00183
85	0.56750	0.09524	0.03362	0.01590	0.00882	0.00541	0.00357	0.00248	0.00179
90	0.55593	0.09331	0.03294	0.01557	0.00864	0.00530	0.00350	0.00243	0.00176
95	0.54465	0.09142	0.03227	0.01526	0.00846	0.00520	0.00343	0.00238	0.00172
100	0.53369	0.08959	0.03162	0.01495	0.00829	0.00509	0.00336	0.00233	0.00169
110	0.51276	0.08607	0.03038	0.01437	0.00797	0.00489	0.00323	0.00224	0.00162
120	0.49322	0.08278	0.02922	0.01382	0.00766	0.00471	0.00310	0.00215	0.00156
130	0.47500	0.07972	0.02814	0.01331	0.00738	0.00453	0.00299	0.00207	0.00150
140	0.45805	0.07686	0.02713	0.01283	0.00712	0.00437	0.00288	0.00200	0.00145
150	0.44227	0.07420	0.02619	0.01238	0.00687	0.00422	0.00278	0.00193	0.00140
160	0.42756	0.07171	0.02531	0.01197	0.00664	0.00408	0.00269	0.00187	0.00135
170	0.41383	0.06940	0.02449	0.01158	0.00642	0.00394	0.00260	0.00181	0.00131
180	0.40100	0.06723	0.02372	0.01122	0.00622	0.00382	0.00252	0.00175	0.00126
190	0.38898	0.06520	0.02301	0.01088	0.00603	0.00370	0.00244	0.00170	0.00123
200	0.37771	0.06330	0.02233	0.01056	0.00586	0.00360	0.00237	0.00165	0.00119
250	0.33045	0.05533	0.01952	0.00923	0.00512	0.00314	0.00207	0.00144	0.00104
300	0.29445	0.04926	0.01737	0.00821	0.00455	0.00280	0.00184	0.00128	0.000925
350	0.26607	0.04447	0.01568	0.00741	0.00411	0.00252	0.00166	0.00115	0.000835
400	0.24309	0.04060	0.01431	0.00676	0.00375	0.00230	0.00152	0.00105	0.000762
450	0.22408	0.03741	0.01318	0.00623	0.00345	0.00212	0.00140	0.000970	0.000702
500	0.20807	0.03471	0.01223	0.00578	0.00320	0.00197	0.00130	0.000900	0.000651
600	0.18254	0.03042	0.01071	0.00506	0.00281	0.00172	0.00114	0.000788	0.000570
700	0.16303	0.02715	0.00956	0.00452	0.00250	0.00154	0.00101	0.000703	0.000508
800	0.14760	0.02456	0.00865	0.00408	0.00226	0.00139	0.000915	0.000636	0.000460
900	0.13505	0.02246	0.00790	0.00373	0.00207	0.00127	0.000837	0.000581	0.000420
1000	0.12463	0.02071	0.00729	0.00344	0.00191	0.00117	0.000771	0.000536	0.000387
1500	0.09094	0.01508	0.00530	0.00250	0.00139	0.000851	0.000561	0.000390	0.000282
2000	0.07236	0.01198	0.00421	0.00199	0.00110	0.000676	0.000445	0.000309	0.000224
3000	0.05214	0.00862	0.00303	0.00143	0.000791	0.000486	0.000320	0.000222	0.000161

**Table 2:** Helium. Excitation energies *E* in eV, dipole *f* values from uncorrelated wave functions (*f*_sc_), *f* values from correlated wave functions (*f*_accu_) by Drake [8], and BE*f*–scaled excitation cross sections *σ*_BE_*_f_* in Å^2^ as functions of incident electron energy *T* in eV. The initial state is 1*s*^2 1^S. The experimental ionization energy *B* = 24.5874 eV has been used in the scaling. The constants *a*, *b*, and *c* of Eq. (5) are included

Final state	1*s*2*p* ^1^P	1*s*3*p* ^1^P	1*s*4*p* ^1^P	1*s*5*p* ^1^P	1*s*6*p* ^1^P	1*s*7*p* ^1^P	1*s*8*p* ^1^P	1*s*9*p* ^1^P	1*s*10*p* ^1^P
*E*	21.218	23.087	23.742	24.046	24.211	24.311	24.375	24.420	24.452
*f*_sc_	0.2583	0.07061	0.02899	0.01466	0.00844	0.00529	0.00354	0.00248	0.00181
*f*_accu_	0.2762	0.07343	0.02986	0.01504	0.00863	0.00541	0.00361	0.00253	0.00184
Const. *a*	0.165601	0.041611	0.016111	0.008298	0.004740	0.002963	0.001975	0.001383	0.001006
Const. *b*	−0.076942	−0.018087	−0.007040	−0.003475	−0.001972	−0.001227	−0.000816	−0.000570	−0.000414
Const. *c*	0.033306	0.002104	−0.000045	−0.000228	−0.000194	−0.000146	−0.000108	−0.000080	−0.000061
*T*	*σ*_BE_*_f_*	*σ*_BE_*_f_*	*σ*_BE_*_f_*	*σ*_BE_*_f_*	*σ*_BE_*_f_*	*σ*_BE_*_f_*	*σ*_BE_*_f_*	*σ*_BE_*_f_*	*σ*_BE_*_f_*
23	0.01939								
24	0.02474	0.00337							
25	0.02933	0.00498	0.00159	0.000684	0.000354	0.000206	0.000130	0.0000879	0.0000621
26	0.03343	0.00625	0.00216	0.000997	0.000543	0.000329	0.000214	0.000148	0.000106
27	0.03716	0.00735	0.00264	0.00125	0.000688	0.000421	0.000277	0.000192	0.000139
28	0.04058	0.00832	0.00305	0.00146	0.000812	0.000500	0.000330	0.000229	0.000166
29	0.04375	0.00921	0.00342	0.00165	0.000922	0.000569	0.000376	0.000262	0.000190
30	0.04669	0.01002	0.00376	0.00182	0.00102	0.000631	0.000418	0.000291	0.000211
35	0.05875	0.01329	0.00510	0.00250	0.00141	0.000878	0.000583	0.000407	0.000295
40	0.06757	0.01565	0.00607	0.00299	0.00169	0.00105	0.000700	0.000489	0.000355
45	0.07413	0.01740	0.00678	0.00335	0.00190	0.00118	0.000787	0.000550	0.000399
50	0.07903	0.01871	0.00731	0.00362	0.00206	0.00128	0.000851	0.000595	0.000432
60	0.08542	0.02043	0.00802	0.00397	0.00226	0.00141	0.000937	0.000655	0.000476
70	0.08883	0.02138	0.00841	0.00417	0.00237	0.00148	0.000985	0.000689	0.000501
80	0.09043	0.02185	0.00861	0.00427	0.00243	0.00152	0.00101	0.000706	0.000513
90	0.09088	0.02202	0.00868	0.00431	0.00245	0.00153	0.00102	0.000713	0.000518
100	0.09060	0.02199	0.00868	0.00431	0.00245	0.00153	0.00102	0.000713	0.000518
110	0.08983	0.02184	0.00862	0.00428	0.00244	0.00152	0.00101	0.000709	0.000515
120	0.08876	0.02161	0.00853	0.00424	0.00242	0.00151	0.00100	0.000702	0.000510
130	0.08748	0.02132	0.00842	0.00419	0.00239	0.00149	0.000991	0.000693	0.000504
140	0.08609	0.02100	0.00830	0.00413	0.00235	0.00147	0.000976	0.000683	0.000497
150	0.08462	0.02066	0.00817	0.00406	0.00231	0.00144	0.000961	0.000672	0.000489
160	0.08313	0.02030	0.00803	0.00399	0.00227	0.00142	0.000945	0.000661	0.000481
170	0.08162	0.01995	0.00789	0.00392	0.00224	0.00139	0.000929	0.000650	0.000472
180	0.08012	0.01959	0.00775	0.00385	0.00220	0.00137	0.000912	0.000638	0.000464
190	0.07864	0.01923	0.00761	0.00378	0.00216	0.00135	0.000896	0.000627	0.000456
200	0.07718	0.01888	0.00747	0.00372	0.00212	0.00132	0.000880	0.000616	0.000448
225	0.07370	0.01805	0.00714	0.00355	0.00202	0.00126	0.000841	0.000589	0.000428
250	0.07046	0.01726	0.00683	0.00340	0.00194	0.00121	0.000805	0.000563	0.000410
275	0.06747	0.01654	0.00655	0.00326	0.00186	0.00116	0.000772	0.000540	0.000393
300	0.06471	0.01587	0.00628	0.00313	0.00178	0.00111	0.000740	0.000518	0.000377
350	0.05982	0.01468	0.00581	0.00289	0.00165	0.00103	0.000685	0.000479	0.000349
400	0.05564	0.01365	0.00541	0.00269	0.00153	0.000957	0.000638	0.000446	0.000324
450	0.05203	0.01277	0.00506	0.00252	0.00144	0.000896	0.000597	0.000417	0.000304
500	0.04890	0.01201	0.00476	0.00237	0.00135	0.000842	0.000561	0.000392	0.000285
600	0.04372	0.01074	0.00425	0.00212	0.00121	0.000753	0.000502	0.000351	0.000255
700	0.03962	0.00973	0.00386	0.00192	0.00109	0.000683	0.000455	0.000318	0.000231
800	0.03628	0.00891	0.00353	0.00176	0.00100	0.000625	0.000416	0.000291	0.000212
900	0.03350	0.00823	0.00326	0.00162	0.000925	0.000578	0.000385	0.000269	0.000196
1000	0.03116	0.00766	0.00303	0.00151	0.000861	0.000537	0.000358	0.000250	0.000182
1500	0.02333	0.00573	0.00227	0.00113	0.000645	0.000402	0.000268	0.000187	0.000136
2000	0.01885	0.00463	0.00183	0.000913	0.000521	0.000325	0.000216	0.000151	0.000110
3000	0.01383	0.00340	0.00135	0.000670	0.000382	0.000238	0.000159	0.000111	0.0000807

**Table 3:** Lithium. Excitation energies *E* in eV, dipole *f* values calculated from uncorrelated wave functions (*f*_sc_), *f* values calculated from correlated wave functions (*f*_accu_) as explained in the text, and BE*f*–scaled excitation cross sections *σ*_BE_*_f_* in Å^2^ as functions of incident electron energy *T* in eV. The experimental ionization energy *B* = 5.3917 eV has been used in the scaling. The constants *a*, *b*, and *c* of Eq. (5) are included

Excitation	2*s*–2*p*	2*s*–3*p*	2*s*–4*p*	2*s*–5*p*	2*s*–6*p*	2*s*–7*p*	2*s*–8*p*	2*s*–9*p*	2*s*–10
*E*	1.848	3.834	4.522	4.837	5.008	5.110	5.177	5.222	5.254
*f*_sc_	0.7685	0.00340	0.00353	0.00217	0.00135	0.000880	0.000601	0.000427	0.000314
*f*_accu_	0.7468	0.00483	0.00428	0.00260	0.00158	0.00101	0.000683	0.000482	0.000353
Const. *a*	5.658148	0.012056	0.010631	0.006113	0.003666	0.002342	0.001580	0.001113	0.000813
Const. *b*	17.288057	0.219978	0.047005	0.018340	0.009244	0.005372	0.003419	0.002319	0.001650
Const. *c*	−0.226058	0.022768	0.011300	0.005517	0.003062	0.001872	0.001229	0.000850	0.000613
*T*	*σ*_BE_*_f_*	*σ*_BE_*_f_*	*σ*_BE_*_f_*	*σ*_BE_*_f_*	*σ*_BE_*_f_*	*σ*_BE_*_f_*	*σ*_BE_*_f_*	*σ*_BE_*_f_*	*σ*_BE_*_f_*
2	14.59367								
2.5	27.24288								
3	33.14707								
3.5	36.69134								
4	38.97838	0.53291							
4.5	40.48711	0.87436							
5	41.47440	0.97533	0.15740	0.04132					
5.5	42.09531	1.00527	0.18668	0.06775	0.03110	0.01653	0.00976	0.00622	0.00421
6	42.45067	1.00592	0.19520	0.07506	0.03657	0.02057	0.01277	0.00851	0.00598
8	42.33337	0.92889	0.18523	0.07364	0.03691	0.02123	0.01341	0.00905	0.00643
10	41.07186	0.83871	0.16733	0.06671	0.03351	0.01930	0.01221	0.00825	0.00586
15	36.87740	0.66670	0.13449	0.05414	0.02736	0.01582	0.01003	0.00679	0.00483
20	32.98349	0.55296	0.11354	0.04625	0.02354	0.01367	0.00869	0.00589	0.00420
25	29.73768	0.47298	0.09886	0.04071	0.02085	0.01215	0.00775	0.00526	0.00375
30	27.06106	0.41366	0.08789	0.03654	0.01881	0.01099	0.00702	0.00478	0.00341
35	24.83566	0.36787	0.07932	0.03324	0.01719	0.01007	0.00645	0.00439	0.00314
40	22.96265	0.33143	0.07241	0.03056	0.01586	0.00932	0.00597	0.00407	0.00291
45	21.36655	0.30171	0.06671	0.02832	0.01475	0.00868	0.00557	0.00380	0.00272
50	19.99071	0.27700	0.06190	0.02643	0.01380	0.00813	0.00522	0.00357	0.00255
60	17.73927	0.23823	0.05425	0.02337	0.01226	0.00725	0.00466	0.00319	0.00228
70	15.97298	0.20918	0.04839	0.02100	0.01106	0.00655	0.00422	0.00289	0.00207
80	14.54840	0.18657	0.04375	0.01911	0.01009	0.00599	0.00386	0.00264	0.00190
90	13.37361	0.16847	0.03998	0.01755	0.00930	0.00553	0.00357	0.00244	0.00175
100	12.38704	0.15364	0.03684	0.01625	0.00863	0.00513	0.00332	0.00227	0.00163
110	11.54596	0.14126	0.03420	0.01515	0.00806	0.00480	0.00310	0.00213	0.00153
120	10.81975	0.13076	0.03193	0.01419	0.00756	0.00451	0.00292	0.00200	0.00144
130	10.18588	0.12175	0.02996	0.01336	0.00713	0.00426	0.00276	0.00189	0.00136
140	9.62741	0.11393	0.02823	0.01263	0.00675	0.00403	0.00261	0.00179	0.00129
150	9.13135	0.10707	0.02671	0.01198	0.00641	0.00383	0.00248	0.00170	0.00123
160	8.68756	0.10101	0.02535	0.01140	0.00611	0.00365	0.00237	0.00163	0.00117
170	8.28799	0.09561	0.02413	0.01087	0.00583	0.00349	0.00226	0.00155	0.00112
180	7.92620	0.09077	0.02303	0.01040	0.00558	0.00334	0.00217	0.00149	0.00107
190	7.59694	0.08641	0.02203	0.00997	0.00536	0.00321	0.00208	0.00143	0.00103
200	7.29592	0.08246	0.02112	0.00958	0.00515	0.00309	0.00200	0.00138	0.000991
225	6.64488	0.07403	0.01917	0.00872	0.00470	0.00282	0.00183	0.00126	0.000907
250	6.10761	0.06719	0.01756	0.00802	0.00433	0.00260	0.00169	0.00116	0.000837
275	5.65611	0.06153	0.01621	0.00743	0.00402	0.00241	0.00157	0.00108	0.000778
300	5.27096	0.05677	0.01507	0.00693	0.00375	0.00225	0.00147	0.00101	0.000727
350	4.64769	0.04920	0.01323	0.00611	0.00331	0.00200	0.00130	0.000894	0.000644
400	4.16411	0.04344	0.01181	0.00548	0.00298	0.00179	0.00117	0.000805	0.000580
450	3.77721	0.03892	0.01068	0.00497	0.00270	0.00163	0.00106	0.000732	0.000528
500	3.46013	0.03526	0.00976	0.00455	0.00248	0.00150	0.000976	0.000673	0.000485
600	2.97030	0.02972	0.00834	0.00391	0.00213	0.00129	0.000842	0.000580	0.000419
700	2.60850	0.02570	0.00730	0.00344	0.00188	0.00114	0.000742	0.000512	0.000369
800	2.32962	0.02267	0.00650	0.00307	0.00168	0.00102	0.000664	0.000458	0.000331
900	2.10765	0.02028	0.00587	0.00278	0.00152	0.000923	0.000603	0.000416	0.000300
1000	1.92652	0.01836	0.00535	0.00254	0.00139	0.000845	0.000552	0.000381	0.000275
1500	1.36023	0.01251	0.00375	0.00179	0.000988	0.000600	0.000393	0.000271	0.000196
2000	1.06067	0.00953	0.00291	0.00140	0.000773	0.000470	0.000308	0.000213	0.000154
3000	0.74561	0.00649	0.00203	0.000984	0.000545	0.000332	0.000218	0.000151	0.000109
